# Design of an Ultra-Wideband MIMO Antenna with Open-Slot Structures for 5G Metal-Frame Smartphones †

**DOI:** 10.3390/s25195973

**Published:** 2025-09-26

**Authors:** Lvwei Chen, Jingjing Bai, Hongliang Gu

**Affiliations:** 1 Yangtze Delta Region Institute (Quzhou), University of Electronic Science and Technology of China (UESTC), Quzhou 324003, China; jingjingbai@csj.uestc.edu.cn; 2 Zhejiang Diverse Communication Technology Co., Ltd., Quzhou 324003, China; jaynu.gu@diversegreat.com

**Keywords:** ultra-wideband, MIMO antenna, open-slot antenna, sub-6 GHz 5G, metal-frame smartphone, high isolation

## Abstract

This paper presents the design and implementation of an ultra-wideband MIMO antenna for sub-6 GHz 5G metal-frame smartphones. The proposed antenna array includes four pairs, each comprising a slotted patch element and an open-slot structure on the metallic rim. The design achieves compactness by sharing the same aperture, critical for overcoming metal-frame smartphone constraints. It minimizes the required ground clearance to 40 × 0.7 mm^2^ to fit the limited space of metallic bezels while maintaining high inter-element isolation. Specifically, one element operates at 2.5–3.8 GHz and 4.8–7.0 GHz, while the other provides continuous coverage from 2.5 to 6.5 GHz, supporting all global sub-6 GHz 5G frequency bands. Specifically, one element operates at 2.5–3.8 GHz and 4.8–7.0 GHz, while the other offers continuous coverage from 2.5 to 6.5 GHz, supporting all sub-6 GHz 5G frequency bands. The open-slot configuration enlarges the operational bandwidth and improves isolation, achieving more than 12.6 dB isolation between elements. A prototype was fabricated and experimentally tested. Measured results indicate that the antenna array maintains a total efficiency above 56% and an envelope correlation coefficient below 0.18 across the target bands. The measured and simulated results are in good agreement, confirming the effectiveness of the proposed design. The proposed antenna is a strong candidate for next-generation 5G smartphone applications due to its wideband performance, high isolation, and compact integration.

## 1. Introduction

5G mobile communications provide significantly enhanced user experiences by offering greater bandwidth, lower latency, and broader coverage [1]. Sub-6 GHz spectrum allocations for 5G vary across regions. For example, the United States has assigned bands around 2.45–2.69 GHz, 3.55–3.7 GHz, and 3.7–4.2 GHz, while China primarily employs 3.3–3.6 GHz and 4.8–5.0 GHz. In addition, the 5G WLAN spectrum extends from 5.15 to 5.85 GHz. These diverse allocations underscore the necessity of designing antennas capable of seamless operation across approximately 2.5–6.0 GHz to fully cover global sub-6 GHz 5G bands.

Multiple-Input Multiple-Output (MIMO) technology [2] is a key enabler of 5G, substantially enhancing channel capacity, spectrum utilization, and system throughput. However, implementing MIMO in compact smartphone platforms introduces significant challenges. The requirement to integrate multiple antennas within a limited physical space often results in reduced isolation between elements, leading to degraded channel capacity and overall data rate. Therefore, advanced decoupling strategies are crucial to maintain high isolation and ensure robust performance in dense smartphone MIMO arrays.

Extensive research has been conducted on 5G smartphone MIMO antenna design. Some reported works cover only a narrow band, such as 3.4–3.6 GHz [3,4,5,6,7,8,9,10,11,12,13,14,15], while others achieve multi-band operation [16,17,18,19,20,21,22,23,24,25,26,27]. However, relatively few designs achieve continuous ultra-wideband performance across the full 2.5–6.5 GHz range, particularly for metal-frame devices.

For instance, the polarization-orthogonal co-frequency dual antenna in [8] is suitable for metal-frame 5G smartphones with isolation above 12 dB. However, it only covers the 3.4–3.6 GHz band, cannot support the capacity enhancement of large-scale MIMO systems, and does not cover the full 2.5–6.5 GHz sub-6 GHz range. The compact building block with two shared-aperture antennas in [10] is designed for metal-rimmed smartphones. It helps reduce the space used by MIMO arrays but offers a bandwidth of less than 1 GHz. It cannot cover the entire 2.5–6.5 GHz band and has large ground clearance, which affects integration in thin devices. The side-edge, frame-printed, eight-port, dual-band antenna array in [22] supports multi-element MIMO operation and enhances integration with the smartphone chassis through its side-mounted design on the non-metallic frame. However, this design provides only fragmented dual-band coverage (3.4–3.6 GHz and 4.8–5.1 GHz), which does not satisfy the need for full 2.5–6.5 GHz sub-6 GHz spectrum support in global 5G applications. Additionally, it is unsuitable for metal-frame 5G smartphones. The dual-band eight-antenna array in [23] enhances 5G channel capacity with a multi-element MIMO design. However, it has inconsistent bandwidth (3.4–3.6 GHz and 5.1–5.9 GHz) and no specific adaptation for metal frames. Metal bezels can easily reduce radiation efficiency and affect practical applications. The ultra-wideband eight-port MIMO antenna array in [28] is designed for metal-frame smartphones and covers 3.3–6.0 GHz. However, it lacks decoupling structures, resulting in isolation only at -11 dB. This low isolation reduces MIMO channel capacity and does not consider the 2.5–3.3 GHz low-frequency band, limiting global sub-6 GHz compatibility. The wideband PIFA-pair-based MIMO antenna in [29] covers 3.3–7.5 GHz with good MIMO diversity performance. However, it is only tested on non-metallic platforms and cannot be used with mainstream metal-frame smartphones. Similarly, the wideband 5G MIMO antenna in [30] is designed for metal-rimmed smartphones with orthogonal-mode dual-antenna pairs. It achieves isolation above 12 dB but only covers 3.3–5.0 GHz. It requires 3 mm ground clearance, which is much larger than the space available in modern slim smartphones. Its feeding network is also complex, increasing PCB routing difficulty and manufacturing costs. The ultra-wideband MIMO antenna system in [31] uses multiple T- and C-shaped slots on metal frames to achieve 3.3–6.0 GHz coverage. However, it needs many etched slots, which increases fabrication complexity and weakens the mechanical strength of metal frames. It can also not be extended to multi-element MIMO systems for higher capacity. A recent wideband antenna pair in [32] is designed for 5G metal-frame smartphones with 1 mm ground clearance to fit ultra-thin devices. However, it is limited to a dual-antenna configuration and cannot be scaled to eight-element MIMO arrays. It also fails to provide clear, full 2.5–6.5 GHz coverage, which affects its adaptability to global sub-6 GHz spectrums.

These studies collectively emphasize the need for a compact wideband MIMO antenna solution that covers 2.5–6.5 GHz with minimal clearance in metal-frame smartphones. Our shared-aperture design is developed to address these limitations effectively. It provides continuous 2.5–6.5 GHz coverage, maintains ground clearance at 40 × 0.7 mm^2^ for compact integration, supports eight-element MIMO operation with isolation above 12.6 dB, and simplifies fabrication. These features make it a more practical solution for next-generation 5G metal-frame smartphones compared to previous works.

Building on this motivation, this paper proposes an eight-element ultra-wideband MIMO antenna array specifically designed for metal-frame 5G smartphones. The array comprises four shared-aperture antenna pairs integrated into the metallic bezel. Each pair is independently fed by two 50 Ω microstrip lines: one excites a slotted patch connected to the frame via a shorting pin, while the other excites open slots etched into the frame. This complementary configuration generates multiple resonant modes, allowing Antenna 1 to cover 2.5–3.8 GHz and 4.8–7.0 GHz, with Antenna 2 providing continuous coverage from 2.5 to 6.5 GHz. Together, they cover all sub-6 GHz 5G bands. Additionally, the open-slot structures enhance bandwidth and reduce inter-element coupling, achieving isolation above 12.6 dB and an envelope correlation coefficient (ECC) below 0.18. A prototype has been fabricated and tested, with measured S-parameters, radiation patterns, and efficiencies strongly aligning with simulations. These results confirm that the proposed design is a promising candidate for next-generation 5G smartphone applications.

In the following sections, first, Section 2 explains the design and analysis of the proposed ultra-wideband MIMO antenna, covering its configuration, operating principles, parametric study, and array layout. Then, Section 3 presents measurement results and analysis, including prototype performance evaluation, radiation pattern verification, and comparisons with prior designs. Section 4 concludes the work by summarizing the antenna’s key features and application value for 5G metal-frame smartphones.

## 2. Design and Analysis of Proposed Antenna

### 2.1. Configuration of Antenna

**Figure 1:**
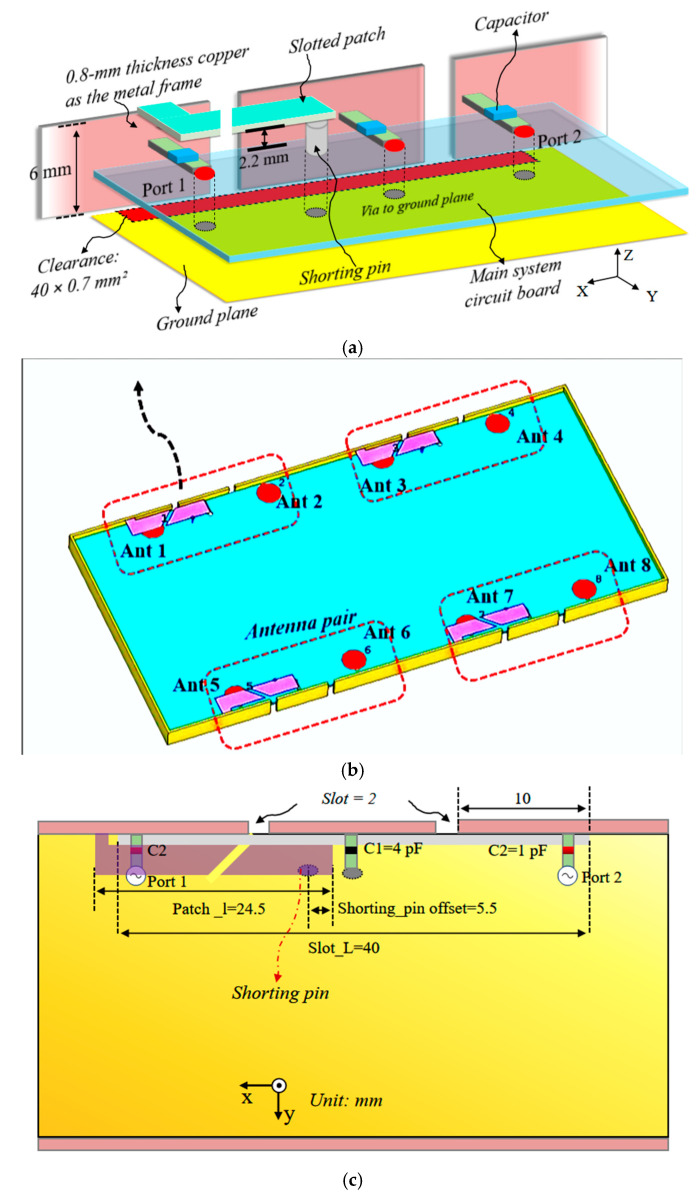
The antenna configuration. (**a**) Antenna pair. (**b**) MIMO antenna array. (**c**) The details of the antenna pair.

Figure 1 illustrates the geometry of the proposed ultra-wideband MIMO antenna array. The antenna is fabricated on an FR4 substrate with a relative permittivity of 4.4, a thickness of 1 mm, and a loss tangent of 0.025, serving as the system printed circuit board (PCB) with overall dimensions of 150 × 75 mm^2^. A metallic frame with a thickness of 0.8 mm and a height of 6 mm. Each antenna pair occupies a ground clearance of 40 × 0.7 mm^2^. In the proposed configuration, four identical antenna pairs (Antennas 1–8) are symmetrically arranged along the longer edge of the PCB. As shown in Figure 1b, each antenna pair comprises a slotted patch connected to the metal frame through a shorting pin linked to the PCB ground and two open slots etched on the frame. Two independent 50 Ω microstrip feeding lines directly excite the metal rim. The detailed dimensions are presented in Figure 1, and the height between the slotted patch and the PCB ground plane is maintained at 2.2 mm.

### 2.2. Antenna Evolution and Analysis

**Figure 2:**
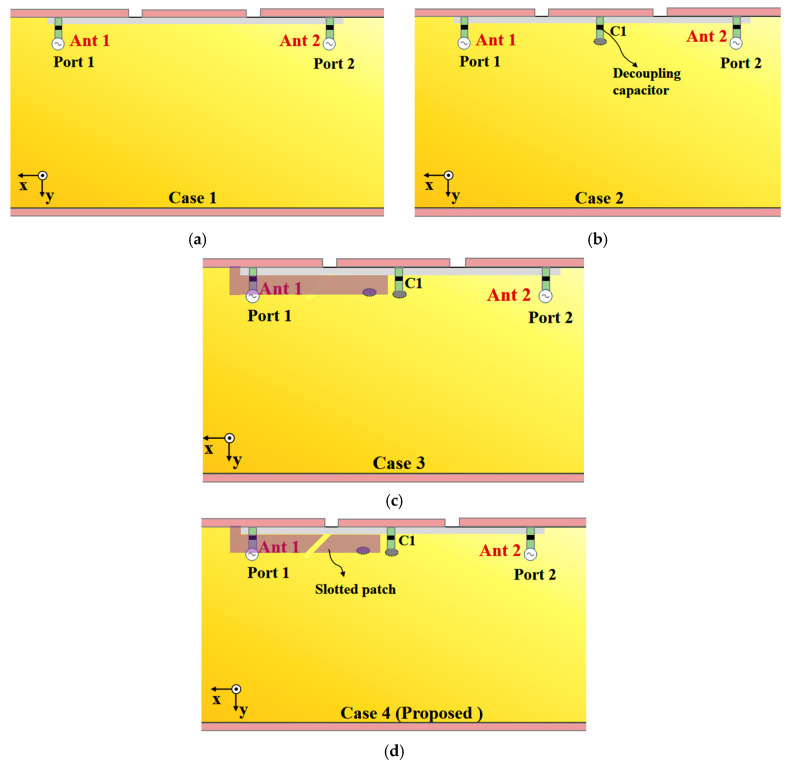

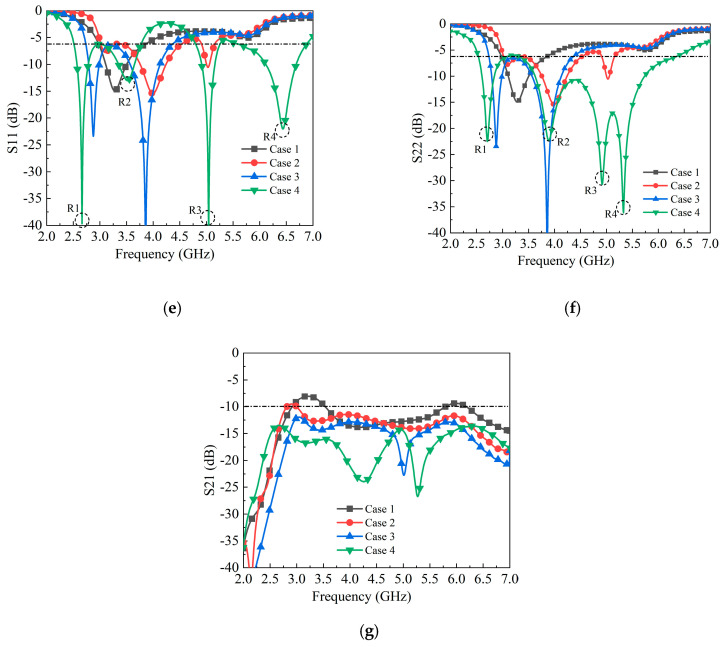
The evolution of the proposed antenna pair and simulated results. (**a**) Case 1. (**b**) Case 2. (**c**) Case 3. (**d**) Case 4 (Proposed). (**e**–**g**) Comparison of S11, S22, and S21 of the antenna pair proposed in Case 1–Case 4.

The design evolution and operating principles of the reference antennas are illustrated in Figure 2. Figure 2a shows the initial topology: two breakpoints are introduced into the metallic bezel, constructing a basic slot-based structure. This configuration inherently excites two fundamental resonant modes: a slot mode and an open-slot mode. However, this preliminary design only provides limited bandwidth and lacks targeted isolation enhancement, failing to meet the dual requirements of wideband coverage and inter-port decoupling for 5G MIMO systems.

To address the isolation and bandwidth limitations of the initial structure, Figure 2b,c incorporate two critical improvements: a decoupling capacitor (C1) and a new metallic patch. The decoupling capacitor (a 0402 multilayer ceramic capacitor (MLCC) with a tolerance of ±5% and a self-resonance frequency above 10 GHz) is integrated between the antenna ports to suppress mutual coupling, effectively boosting the isolation performance of the grounded shared-aperture antenna pair. Meanwhile, the added metallic patch enhances the coupling effect between the antenna and the metallic bezel, which induces an additional resonant component to extend the upper-frequency coverage.

Building on the design in Figure 2b,c further optimizes the metallic patch structure to strengthen the correlation between the patch and the slotted bezel. This adjustment improves the stability of the antenna’s resonant modes. However, it still fails to fully cover the 2.5–6.5 GHz target band, as a bandwidth gap persists in the mid-frequency range, restricting its applicability to 5G scenarios.

To eliminate this mid-frequency gap and realize continuous coverage of the 2.5–6.5 GHz sub-6 GHz band, Figure 2d proposes a slotted-patch modification: precise slots are etched into the metallic patch of Figure 2c’s configuration, forming a slotted-patch structure. Introducing these slots alters the current distribution on the patch surface, generating additional resonant modes that complement the existing slot and open-slot modes. This synergistic effect effectively bridges the mid-frequency gap, enabling the antenna to achieve a continuous impedance bandwidth covering the 2.5–6.5 GHz target band.

**Figure 3:**
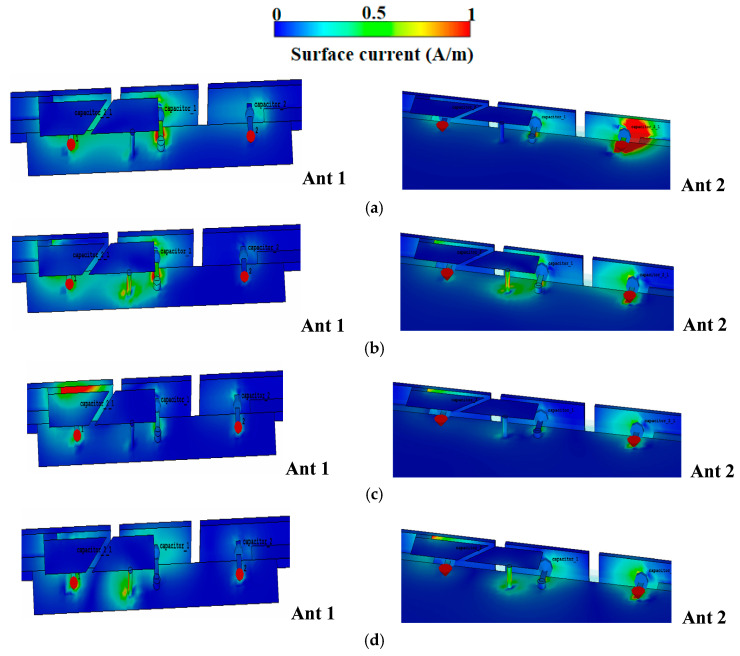
The current distribution of the proposed Ant 1 and Ant 2. (**a**) 2.8 GHz. (**b**) 3.5 GHz. (**c**) 5.0 GHz. (**d**) 5.5 GHz.

The asymmetric antenna pair in Figure 2 closes the 4.0–5.0 GHz gap via two distinct categories of open-slot modes: (1) Modes R1 and R3 are introduced and established between the metallic bezel and the PCB, and (2) Modes R2 and R4 are generated through the coupling among the slotted patch, the PCB, the shorting pins, and the metallic bezel. As shown in Figure 3, R1 and R3 originate from the open slot between the metallic bezel and the PCB, while R2 and R4 stem from the open slot among the slotted patch, the PCB, the shorting pins, and the metallic bezel. The combined bandwidths of these modes eliminate the interval gap in the 4.0–5.0 GHz range.

**Figure 4:**
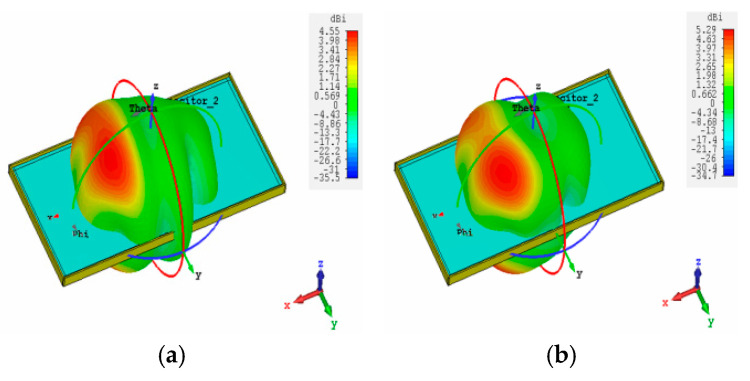

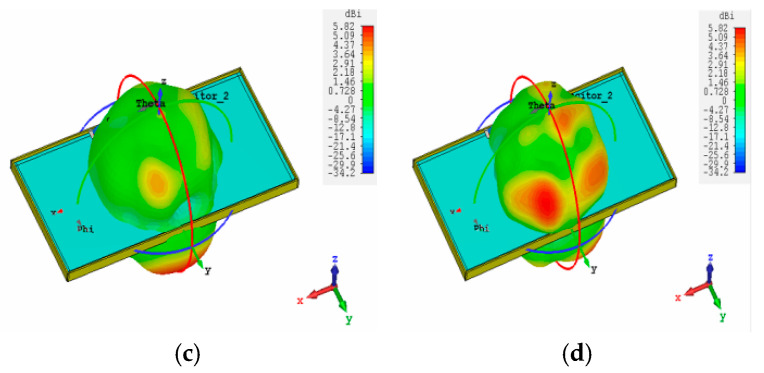
The radiation pattern of the proposed Ant 1. (**a**) 2.8 GHz. (**b**) 3.5 GHz. (**c**) 5.0 GHz. (**d**) 6.2 GHz.

Figure 4 presents simulated three-dimensional radiation patterns further to evaluate the radiation characteristics of the proposed antenna pair. As illustrated in Figure 4a,b, the main lobes are oriented along the +x-axis at the lower frequencies of 2.8 GHz and 3.6 GHz. In contrast, at higher operating frequencies, the radiation patterns shift toward the +y-axis. This behavior indicates that the principal radiation directions of the lower and higher bands are mutually orthogonal, which is a key feature supporting polarization diversity and spatial isolation in multi-band antenna systems.

### 2.3. Parametric Study

It is essential to investigate how key design parameters influence the performance of the proposed antenna pair. To achieve optimal performance, several critical parameters and their effects are analyzed.

**Figure 5:**
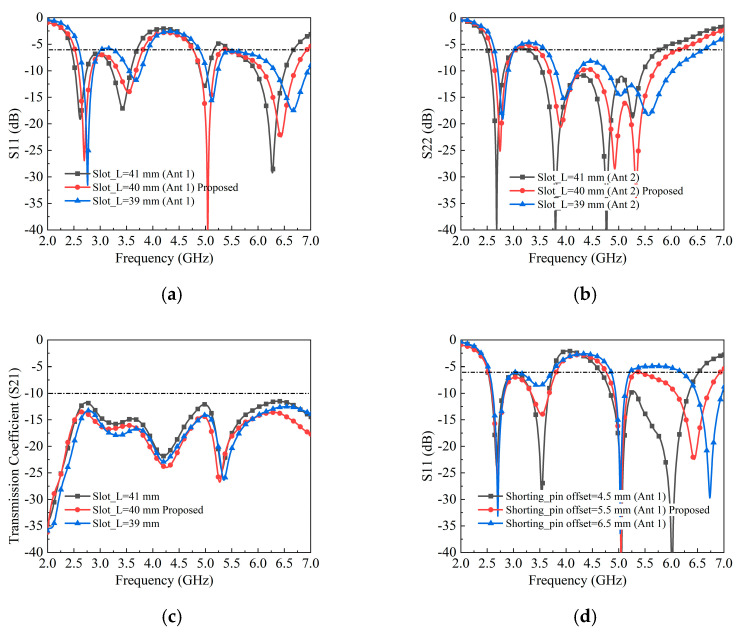

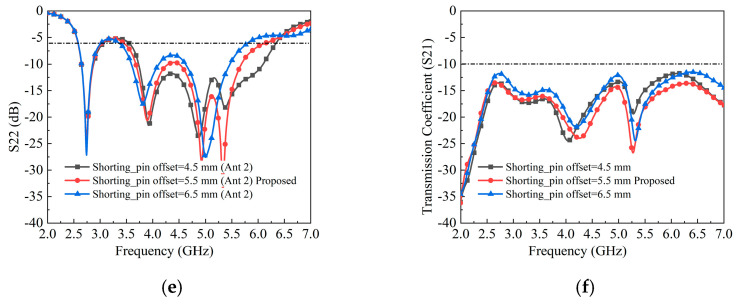
Simulated S-parameters results of different parameters. (**a**–**c**) The length of Slot_L. (**d**–**f**) The location of the shorting pin.

First, the influence of the slot length (slot_L) is evaluated, as illustrated in Figure 5. When slot_L increases from 39 mm to 41 mm, all four resonant modes of Antenna 1 and Antenna 2 shift toward lower frequencies, as shown in Figure 5a,b. However, variations in slot_L have only a minor impact on the isolation performance of the antenna pair.

Second, the effect of the shorting-pin position is studied, and the simulated results are presented in Figure 5. It is evident that increasing the shorting-pin offset from 4.5 mm to 6.5 mm shifts the second and fourth resonant modes for both antennas, while the first and third modes remain nearly unchanged. This behavior can be explained by the resonance mechanisms: the first and third modes are mainly generated by the open structure formed between the metal rim and the PCB ground, whereas the second and fourth modes arise from the slotted patch and the PCB ground. Furthermore, the shorting-pin offset significantly affects the isolation performance, indicating that the slotted patch plays an important role in suppressing mutual coupling between the two antenna elements.

### 2.4. MIMO Antenna Array

**Figure 6:**
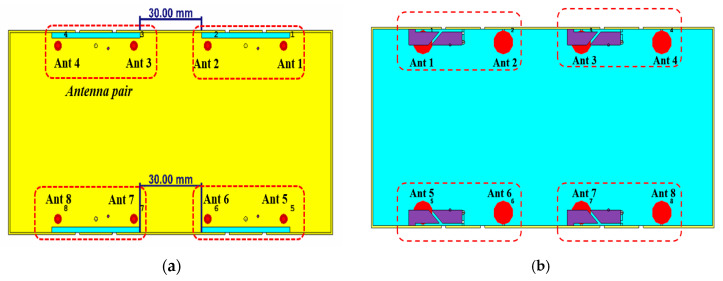
The proposed 8 × 8 antenna array. (**a**) Front view. (**b**) Back view.

**Figure 7:**
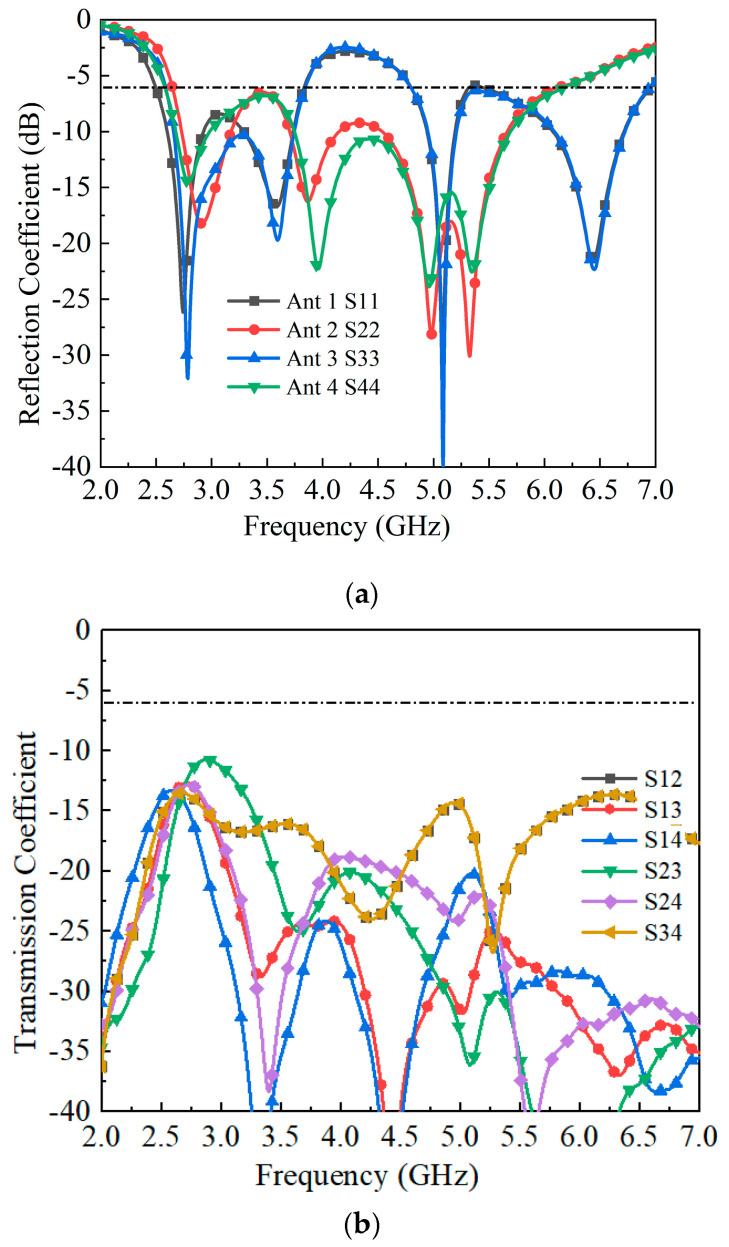
Simulated S-parameter results of the proposed antenna array. (**a**) S-parameters. (**b**) The isolation between each antenna.

**Figure 8:**
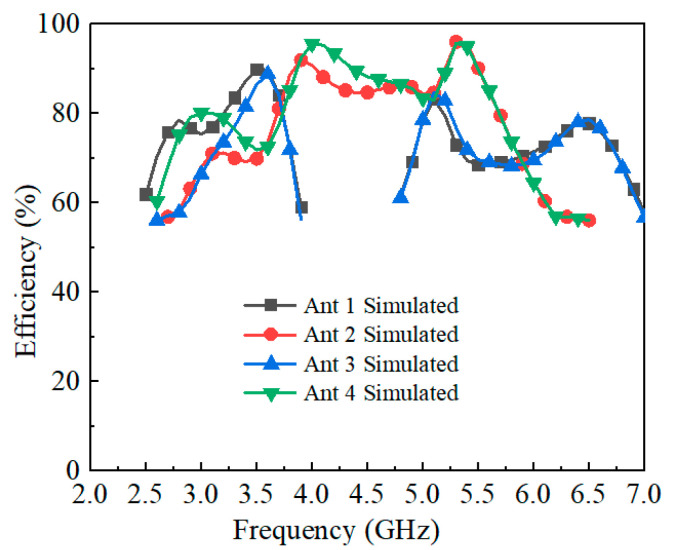
Simulated total efficiencies of the proposed antenna array.

An eight-element MIMO antenna array is developed to further enhance channel capacity by integrating four antenna pairs, as illustrated in Figure 6. The array adopts a symmetrical configuration, with a spacing of 30 mm between adjacent antenna pairs. The simulated reflection coefficients and isolation characteristics are presented in Figure 7. The minimum isolation, observed between Antennas 2 and 3, is better than −11.6 dB, which remains acceptable for 5G smartphone applications. The simulated total efficiencies are summarized in Figure 8: Antennas 1 and 3 achieve efficiencies exceeding 59% and 61% in their dual operating bands, while Antennas 2 and 4 maintain efficiencies above 56% across the working frequency range.

## 3. Measurement Results

This section presents the construction of the eight-element MIMO antenna system, the fabrication of a prototype, and the evaluation of its performance, including the effects of user interaction. In addition, a comparison with previously reported designs is provided to highlight the advantages of the proposed approach.

### 3.1. MIMO Performance

**Figure 9:**
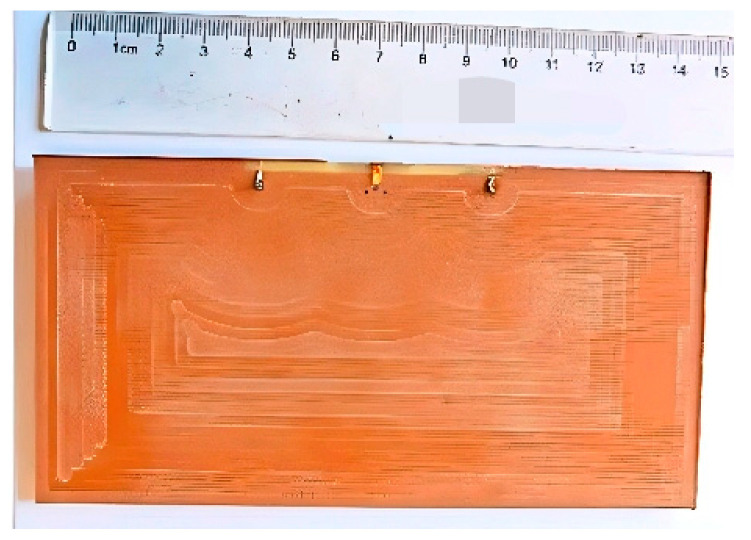
Photos of the fabricated antenna.

**Figure 10:**
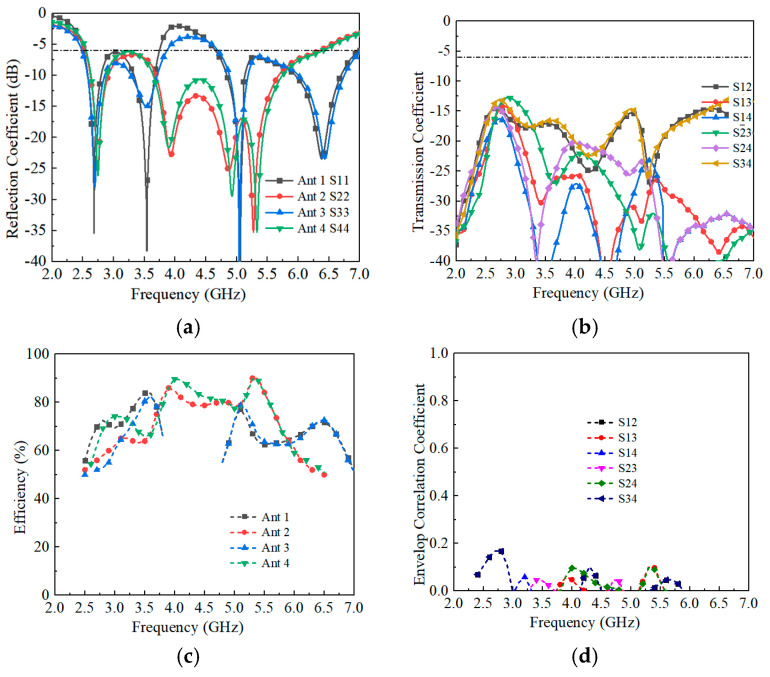
The measured results of the proposed antenna array. (**a**) Reflection coefficients. (**b**) Transmission coefficients. (**c**) Total efficiencies. (**d**) ECCs.

The fabricated prototype is shown in Figure 9, and the corresponding measured S-parameters are plotted in Figure 10. Due to the symmetry of the array, only results for Antennas 1–4 are reported. The measured 6 dB impedance bandwidth of Antenna 1 spans 2.5–3.8 GHz and 4.8–7.0 GHz, while Antenna 2 achieves continuous coverage from 2.5 to 6.5 GHz. As shown in Figure 10b, the measured isolation between antenna elements is consistently better than 12.6 dB across the entire operating band. Minor discrepancies between simulated and measured results are observed, which can be attributed to fabrication tolerances and connector (SMA) losses, the latter typically contributing to slightly higher measured isolation.

The total radiation efficiencies are summarized in Figure 10c. Antennas 1 and 3 achieve efficiencies above 59% and 61% in their dual operating bands, respectively, while Antennas 2 and 4 maintain efficiencies above 56% across the 2.5–6.5 GHz range. The envelope correlation coefficient (ECC), a key metric for evaluating MIMO performance, was calculated from the measured S-parameters [33,34]. As shown in Figure 10d, the measured ECC remains below 0.2 throughout the band of interest, well within the commonly accepted threshold of 0.5. This confirms that the proposed antenna array exhibits excellent diversity performance and effectively suppresses correlation between antenna elements.

### 3.2. Radiation Performance

**Figure 11:**
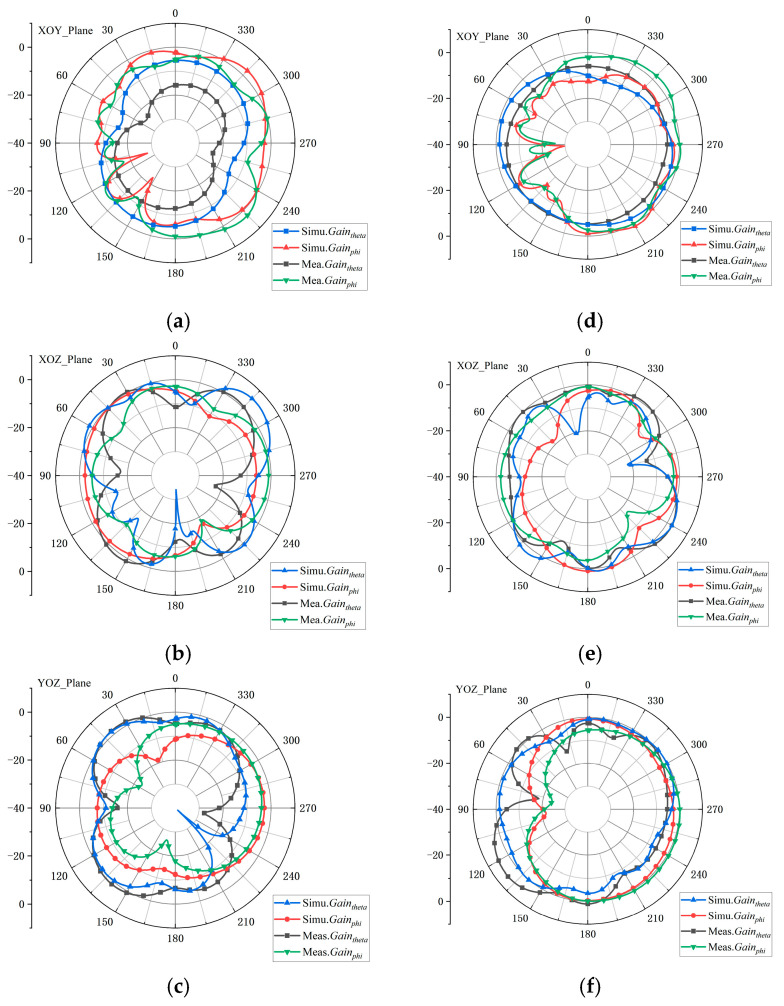
Simulated and measured radiation patterns of Ant 1 at 3.5 and 5 GHz. (**a**) 3.5 GHz at XOY plane. (**b**) 3.5 GHz at XOZ plane. (**c**) 3.5 GHz at YOZ plane. (**d**) 5.0 GHz at XOY plane. (**e**) 5.0 GHz at XOZ plane. (**f**) 5.0 GHz at YOZ plane.

The simulated and measured radiation patterns at 3.5 GHz are presented in Figure 11, where only the results for Antennas 1 and 2 are shown due to the structural symmetry. The measured patterns agree closely with the simulations, confirming the accuracy of the design.

### 3.3. Hand and Head Effects

**Figure 12:**
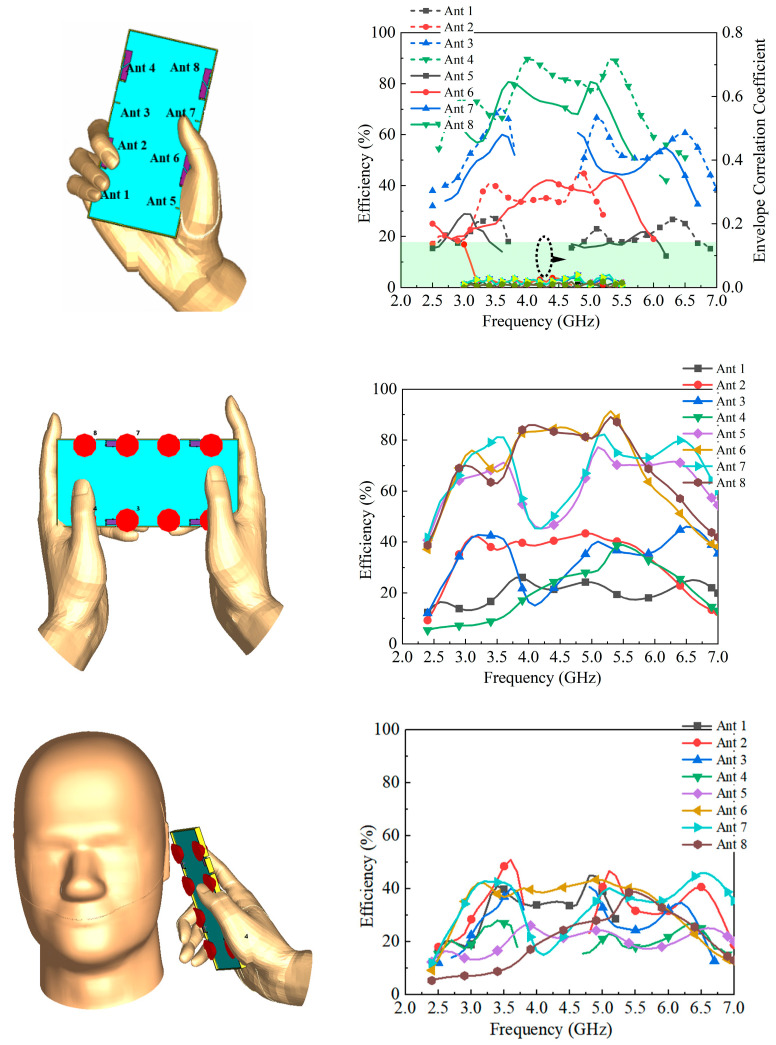
The simulated results of hand and head grip.

The effect of user interaction on the proposed MIMO antenna array was also investigated. As illustrated in Figure 12, the antenna array was modeled in a hand-held, two-hand-held hold, and a call scenario, and the simulated total efficiencies of all eight antennas were evaluated [35]. The results indicate that the efficiencies of Antennas 4 and 8 remain nearly unchanged, whereas those of Antennas 1, 2, 5, and 6 drop to below 45% compared with the free-space case. This degradation is attributed to the hand acting as a lossy dielectric medium that absorbs radiated energy and reduces antenna performance. As shown in Figure 12, the proposed antenna’s ECC remains below 0.25 throughout the 2.5–6.5 GHz sub-6 GHz band of interest. This value is well below the industry threshold of 0.5 for 5G MIMO antenna systems, ensuring minimal signal correlation between adjacent elements and stable multi-channel transmission performance.

### 3.4. Comparison

To emphasize the novelty and advantages of the proposed design, Table 1 compares the results of this work with other recently reported 5G smartphone MIMO antennas. Most previously published wideband arrays, such as those in [15,25,26,29], lack compatibility with metal-frame smartphones, which restricts their industrial applicability. For instance, the wideband (3.3–7.5 GHz) antenna presented in [29] cannot be implemented on metal-rimmed platforms. In contrast, the proposed array not only achieves a wider and continuous operating bandwidth compared to all designs listed in Table 1 but also enables full metal-frame integration. Additionally, it maintains superior compactness with a clearance requirement of only 0.7 mm, which is more compact than the 3 mm clearance adopted in [28,30]. Meanwhile, the proposed array exhibits higher total efficiency exceeding 56%, outperforming the >40% efficiency in [28] and >31.6% efficiency in [30]. Furthermore, the isolation performance remains above 12.6 dB, satisfying the 5G MIMO systems requirements. This addresses the key challenge of realizing wideband, efficient, and practically deployable 5G MIMO antennas for metal-frame smartphone applications.

## 4. Conclusions

An eight-element, ultra-wideband MIMO antenna array for sub-6 GHz 5G communications is presented. The design utilizes slotted patches and open-slot structures, which synergistically broaden the operating bandwidth and suppress mutual coupling between antenna elements. As validated by both simulations and measurements, the proposed array achieves isolation levels exceeding 12.6 dB across the 2.5–6.5 GHz band, maintains radiation efficiency above 56%, and ensures low ECC values below 0.18. Compared with state-of-the-art designs summarized in Table 1, the proposed array not only enables full compatibility with metal-frame smartphone integration but also delivers a wider continuous operating bandwidth, higher radiation efficiency, and more compact structural reuse. Specifically, Table 1 demonstrates that the proposed array outperforms referenced works in terms of total bandwidth coverage (2.5–6.5 GHz for Ant 2, surpassing discrete or narrower bands in [25,30], etc.), efficiency (>56% vs. >31.6% in [30]), and metal-frame adaptability. The strong agreement between simulated and measured results further validates the design’s effectiveness. Therefore, the proposed broadband MIMO antenna array addresses critical challenges in 5G smartphone antenna design and represents a promising solution for future 5G mobile applications and 6G communication [36].

## Figures and Tables

**Table 1:** Comparison between the proposed antennas and references.

Ref.	Metal Frame	Clearance (mm)	Total Bandwidth (GHz)	ECC	Isolation (dB)	Total Efficiency (%)
[15]	No	-	0.2 (3.4–3.6)	<0.14	>11.0	>51
[25]	No	-	0.8 + 0.8 (3.1–3.9 & 5.5–6.3)	<0.03	>12.0	>70
[26]	No	-	0.3 + 0.6 (3.3–3.6 & 4.4–5.0)	<0.18	>12.8	>68
[28]	Yes	3	2.7 (3.3–6.0)	<0.1	>11	>40
[29]	No	-	4.2 (3.3–7.5)	<0.05	>10	>40
[30]	Yes	3	1.7 (3.3–5.0)	<0.11	>12	>31.6
Prop.	Yes	0.7	Ant 1: 1.3 + 2.2 (2.5–3.8 & 4.8–7.0)	<0.18	>12.6	>56
			Ant 2: 4 (2.5–6.5)	<0.18	>12.6	>56

## Data Availability

Data are contained within the article.
